# Toward a conceptual framework for managing and conserving marine habitats: A case study of kelp forests in the Salish Sea

**DOI:** 10.1002/ece3.8510

**Published:** 2022-01-12

**Authors:** Jordan A. Hollarsmith, Kelly Andrews, Nicole Naar, Samuel Starko, Max Calloway, Adam Obaza, Emily Buckner, Daniel Tonnes, James Selleck, Thomas W. Therriault

**Affiliations:** ^1^ Alaska Fisheries Science Center National Marine Fisheries Service National Oceanic and Atmospheric Administration Seattle Washington USA; ^2^ Department of Biological Sciences Simon Fraser University Burnaby British Columbia Canada; ^3^ Pacific Biological Station, Fisheries and Oceans Canada Nanaimo British Columbia Canada; ^4^ Conservation Biology Division Northwest Fisheries Science Center National Marine Fisheries Service National Oceanic and Atmospheric Administration Seattle Washington USA; ^5^ Washington Sea Grant College of the Environment University of Washington Seattle Washington USA; ^6^ Department of Biology University of Victoria Victoria British Columbia Canada; ^7^ Aquatic Resources Division Washington Department of Natural Resources Nearshore Habitat Program Olympia Washington USA; ^8^ Paua Marine Research Group San Diego California USA; ^9^ Puget Sound Restoration Fund Bainbridge Island Washington USA; ^10^ West Coast Region National Marine Fisheries Service National Oceanic and Atmospheric Administration Seattle Washington USA

**Keywords:** Drivers–Pressures–State–Impact–Response, ecosystem‐based management, global change, local ecological knowledge, Puget Sound, resource management

## Abstract

Kelp forests are in decline across much of their range due to place‐specific combinations of local and global stressors. Declines in kelp abundance can lead to cascading losses of biodiversity and productivity with far‐reaching ecological and socioeconomic consequences. The Salish Sea is a hotspot of kelp diversity where many species of kelp provide critical habitat and food for commercially, ecologically, and culturally important fish and invertebrate species. However, like other regions, kelp forests in much of the Salish Sea are in rapid decline. Data gaps and limited long‐term monitoring have hampered attempts to identify and manage for specific drivers of decline, despite the documented urgency to protect these important habitats. To address these knowledge gaps, we gathered a focus group of experts on kelp in the Salish Sea to identify perceived direct and indirect stressors facing kelp forests. We then conducted a comprehensive literature review of peer‐reviewed studies from the Salish Sea and temperate coastal ecosystems worldwide to assess the level of support for the pathways identified by the experts, and we identified knowledge gaps to prioritize future research. Our results revealed major research gaps within the Salish Sea and highlighted the potential to use expert knowledge for making informed decisions in the region. We found high support for the pathways in the global literature, with variable consensus on the relationship between stressors and responses across studies, confirming the influence of local ecological, oceanographic, and anthropogenic contexts and threshold effects on stressor–response relationships. Finally, we prioritized areas for future research in the Salish Sea. This study demonstrates the value expert opinion has to inform management decisions. These methods are readily adaptable to other ecosystem management contexts, and the results of this case study can be immediately applied to kelp management.

## Graphical abstract

Data gaps and limited long‐term monitoring have hampered attempts to identify and manage for specific drivers of decline across many ecosystems, despite often documented urgency to protect important habitats. To address these knowledge gaps, we gathered a focus group of experts paired with a comprehensive literature review to identify perceived direct and indirect stressors facing a threatened ecosystem. Our results revealed local research gaps and highlighted the potential to use expert knowledge for making informed decisions in the region.

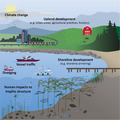

## 1 INTRODUCTION

Coastal marine ecosystems are experiencing unprecedented changes due to climate variability and other human activities (e.g., vessel traffic, upland and nearshore development, and alterations of trophic structure), posing a significant challenge for resource managers and decision makers (Crain et al., 2009; Harley et al., 2006; Hewitt et al., 2016). Species found in shallow coastal environments can be especially vulnerable to the cumulative effects of human modifications to the environment, despite adaptations to disturbance often observed in variable nearshore regions (Crain et al., 2008; Jordan et al., 2009; Peterson & Lowe, 2009; Thrush et al., 2021). These coastal environments often provide critical habitat for ecologically, economically, and culturally important species; therefore, effective management to assure the sustainability of these habitats and the ecosystem services they provide is paramount (Erlandson et al., 2015).

**FIGURE 1:**
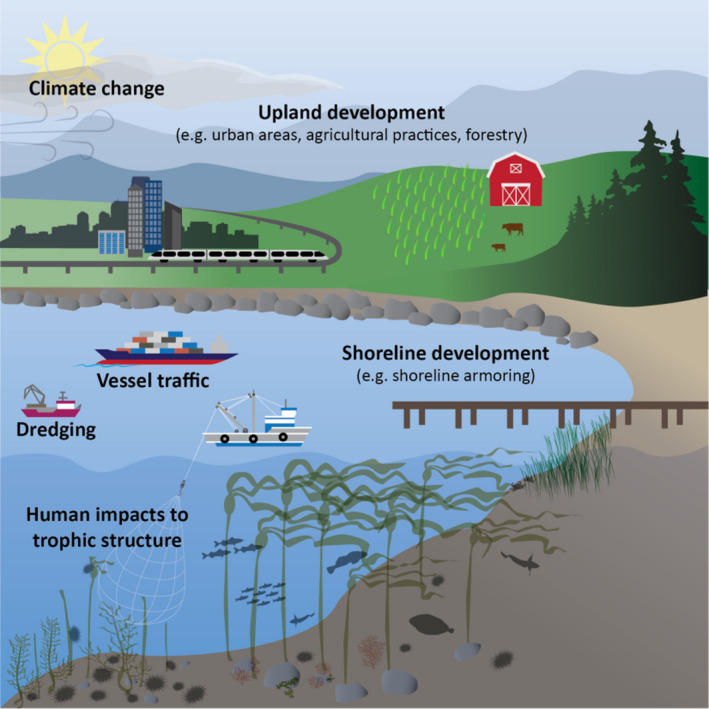
Stressors impacting nearshore kelp forest ecosystems. Figure art by Su Kim

**FIGURE 2:**
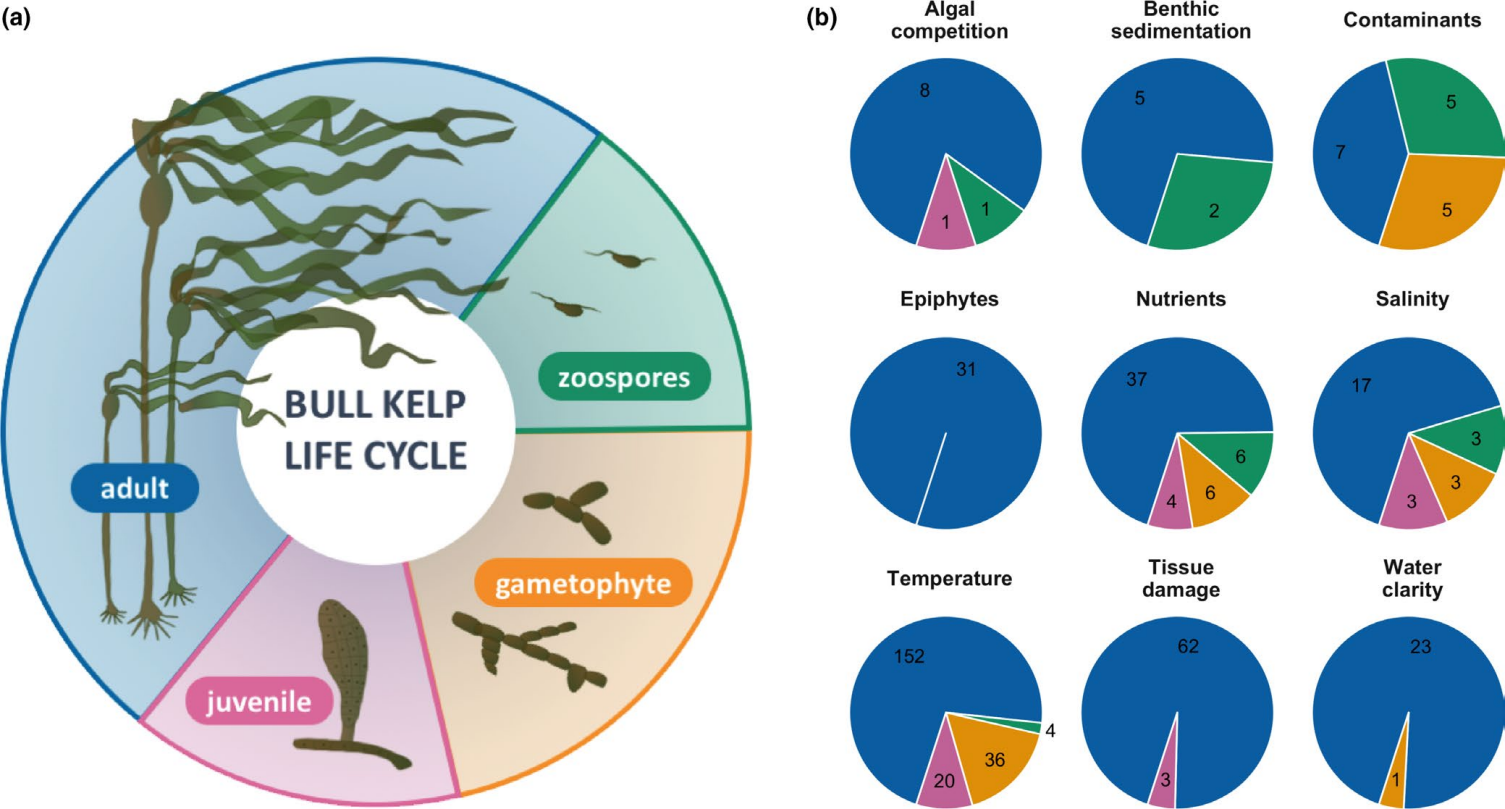
(a) Bull kelp life cycle, and (b) the proportion of studies identified by stressor and life stage (green represents zoospore, orange—gametophyte, pink—juvenile sporophyte, and blue—adult sporophyte). Numbers in each pie chart indicate the number of studies found

Kelp forests are among these important coastal ecosystems that provide critical ecosystem services (e.g., carbon sequestration, primary productivity, erosion control) and habitat for important life stages of fishes, invertebrates, and marine mammals (Calloway et al., 2020; Duggins et al., 1989; Krause‐Jensen & Duarte, 2016; Teagle et al., 2017). In recent decades, kelp forest ecosystems have suffered widespread declines across much of their range (Filbee‐Dexter & Wernberg, 2018; Krumhansl et al., 2016; Smale, 2020; Wernberg et al., 2019). The drivers of these declines differ by place and include climate change‐amplified marine heatwaves, eutrophication, altered trophic structures, and shoreline development, among other anthropogenic stressors (Bischof et al., 2019; Halpern et al., 2019; Rogers‐Bennett & Catton, 2019; Smale, 2020; Figure 1). These drivers can affect multiple life‐history stages of kelps and may interact to reduce growth, reproduction, and survival of individual kelps and their populations. The impacts of these stressors may also depend on the strength and timing of the impacts and the functional role of different kelp species in the ecosystem: While some species float toward the surface and create upright, buoyant canopies, others remain close to the benthos. Additionally, kelps have a biphasic life history composed of micro‐ and macroscopic stages, each of which may respond differently to stressors (Figure 2a). Regardless of which functional groups make up a given kelp forest, the macroscopic stages create complex, three‐dimensional habitats that form the structural and energetic bases for an abundance of life (Teagle et al., 2017). Declines in kelp populations can therefore have large and cascading impacts on ecological and human communities (Graham, 2004; Shaffer et al., 2020).

A region of particularly high kelp species diversity is the Salish Sea (Druehl, 1970), a fjordal system of inland waterways straddling Washington State (U.S.) and British Columbia (Canada). There have been 21 species of kelp identified within this region, with the bull kelp (*Nereocystis luetkeana*) as the primary floating canopy‐forming species, while the majority of species lie within a few meters of the bottom. Most kelps in this region grow as small forests along a narrow depth band near the shore where they are exposed to large seasonal swings in temperature and salinity. These kelp forests provide critical habitat for threatened or endangered fish and invertebrate species, including Pacific salmon (*Oncorhynchus* spp.), rockfish (*Sebastes* spp.), herring (*Clupea pallasii*), and abalone (*Haliotis kamtschatkana*) (NMFS, 2005, 2014). Recently quantified declines in the extent of kelp forests in Puget Sound raised concerns regarding the availability of critical habitat for these threatened species which motivated the creation of the Puget Sound Kelp Conservation and Recovery Plan (Berry et al., 2021; Calloway et al., 2020). Although the drivers of the declines remain unclear, they are likely the result of cumulative effects from multiple natural and human stressors on the system such as increasing sea surface temperatures and incidences of marine heatwaves (Iwabuchi & Gosselin, 2019; Masson & Cummins, 2007), changes to watersheds and nearshore terrestrial environments (Hansen et al., 2013), and changes to marine ecological communities (Pietsch & Orr, 2015; Zier & Gaydos, 2016). Mapping efforts in other regions of the Salish Sea found kelp population trends were stable or slightly declining, suggesting that stressor intensity and impact varies across basins (Pfister et al., 2018; Schroeder et al., 2020), but differences in the spatial and temporal scales of these studies make comparisons difficult.

The level of data required to quantitatively model the cumulative impacts of multiple stressors on ecosystems such as kelp forests can rapidly surpass available resources (Foley et al., 2017). To overcome this challenge, expert knowledge is increasingly being used as a valuable data source in modeling ecosystem processes, answering management questions, and forecasting the impacts of disturbance. For example, Reum et al. (2019) used diverse expert and stakeholder input to assess management options to rebuild a collapsing fishery in the presence of ongoing climate change; and Stier et al. (2017a, 2017b) quantified how perceptions of food webs based around Pacific herring differed among scientific, local, and traditional knowledge experts. Expert knowledge is an especially valuable data source when modeling complex systems with interacting stressors for which there is little experimental or observational data to build purely quantitative models (McBride & Burgman, 2012). When used in conjunction with quantitative approaches, expert knowledge can guide future research so that limited available resources can focus on the most critical data needs. In addition to modeling complex ecological processes in data‐poor systems, this approach builds communication among stakeholders and increases transparency in decision‐making processes. This is critical because increased stakeholder participation in management decisions promotes support for management actions and successful implementation, as was seen in the design and implementation of marine protected areas in California (Fletcher et al., 2014).

One way to organize conceptual and empirical understandings of complex coastal ecosystems is the DPSIR (Drivers–Pressures–State–Impact–Response) framework (Lewison et al., 2016). The DPSIR framework links ultimate and proximate causes to changes in state variables and allows resource managers to assess the relative impacts and responses of potential management strategies (Turner, 2000). The main components of the model are as follows: (1) Drivers—human activities with an environmental effect (indirect stressors); (2) Pressures—direct positive and negative effects of the Drivers on the environment (direct stressors); (3) State—the condition of the environment; (4) Impact—the effect of the Pressures, measured as the change in State; and (5) Response—policies, interventions, or management priorities adopted to improve the State (Kristensen, 2004). A major strength of the DPSIR methodology is its flexibility, which allows for the use of quantitative data, when available, or expert opinions in the absence of quantitative data. The DPSIR framework has been used to organize understandings, identify research needs, and support management decisions in a number of complex social–ecological systems (Lewison et al., 2016), including recent applications to global microplastic pollution (Miranda et al., 2020), fisheries management in Kenya (Dzoga et al., 2020), and ecotourism in Thailand (Suursaar & Kornpiphat, 2021).

In an effort to fill existing knowledge gaps for Salish Sea kelp ecosystems to inform management decision‐making, we undertook a multistep process. First, we brought together a group of diverse experts from academic institutions and federal, regional, and Indigenous governments in Washington and British Columbia to map the direct and indirect stressors believed to be contributing to kelp decline in the Salish Sea. We used a modified DPSIR framework to organize how experts identified direct and indirect stressors on kelp populations. We then conducted a comprehensive literature review of each stressor identified by the experts, focusing on both regional research in the Salish Sea and related work in global temperate marine ecosystems. In the course of the literature review, we identified research gaps and limitations in local data to guide and prioritize future research efforts. The development of these linkages and the information from the literature review could help drive subsequent semiquantitative analyses, such as qualitative network models or Bayesian belief networks, that evaluate how important each direct and indirect linkage is between Drivers, Pressures, and the State of kelp populations (Hollarsmith et al., 2021). By combining both expert opinion and a comprehensive and structured literature review, we were able to create a robust analysis to inform management despite local data gaps.

## 2 METHODS

### 2.1 Expert‐based conceptual model

We convened a focus group of experts from Washington State (U.S.) and British Columbia (Canada) to develop a conceptual diagram of direct and indirect threats facing kelp ecosystems in the Salish Sea. We first identified experts by contacting researchers, resource managers, and other stakeholders who contributed to the Puget Sound Kelp Conservation and Recovery Plan, and subsequently relied on snowball sampling to invite other experts. The final focus group consisted of 14 invited researchers and resource managers and included participants from NOAA’s West Coast Region and Northwest Fisheries Science Center, Washington Department of Natural Resources, Samish Indian Nation, Puget Sound Restoration Fund, Province of British Columbia Marine Planning Partnership, Parks Canada, Washington Marine Resources Committee, Simon Fraser University, University of Washington, and University of Victoria.

Through a moderated hybrid discussion (in‐person in Mukilteo, WA, or by videoconference), we asked the full group of participants a set of questions to identify the kelp species and indicator of interest and the direct and indirect stressors facing kelp populations in the Salish Sea. The discussion questions were based on the DPSIR framework (Kristensen, 2004; Turner, 2000), but focused only on the Drivers, Pressures, and State components. Specifically, we asked participants the following:

1. What species and life stage are we considering as a management target (i.e., the relevant State)?
2. What are the direct stressors (human or natural) that drive change in kelp populations (i.e., Pressures on kelp)?
3. What are the indirect stressors (human or natural) that drive change in kelp populations (i.e., Drivers of the pressures)?
4. What are the interactions/connections between these direct and indirect stressors with kelp populations?

**FIGURE 3:**
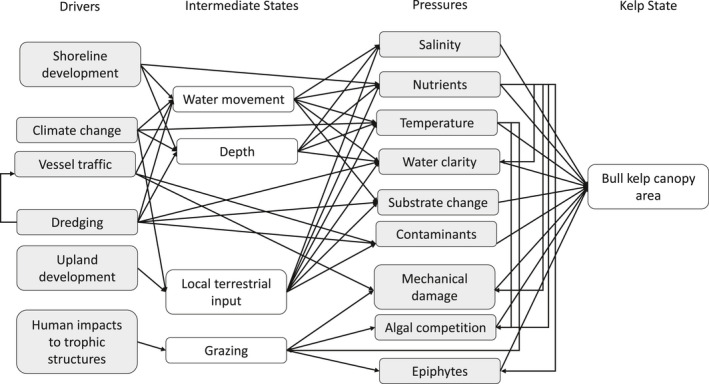
Conceptual diagram of drivers and pressures impacting kelp identified by the focus group of experts

JH moderated the focus group by posing the questions to the group and sketching the developing conceptual diagram on a white board, while NN transcribed the discussion in real time. We used a consensus‐based decision‐making approach to determine the species and life stage focus of kelp for the purpose of the conceptual diagram and to determine which stressors were most important. All participants were invited to respond to each question until no additional indicators/stressors were identified by the group. Any emerging disagreements or refinements were discussed until all focus group members were satisfied with the conceptual diagram. The final list of answers to these questions and the resulting conceptual diagram were used to develop a conceptual model showing the interactive pathways between indirect and direct stressors on kelp populations in the Salish Sea (Figure 3).

### 2.2 Literature review

**FIGURE 4:**
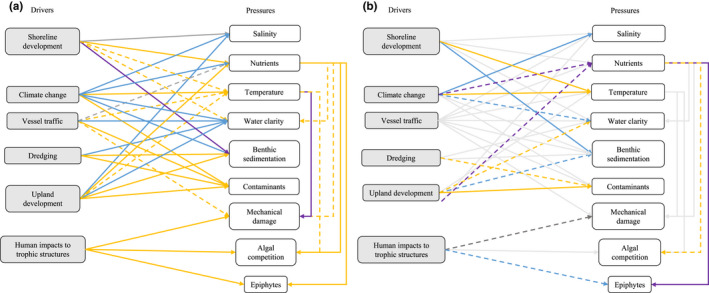
Results of the literature search based on a simplified conceptual diagram, including results for (a) broader coast literature and (b) Salish Sea literature. Color indicates the direction of the relationship (blue represents negative, dark gray—neutral, orange—positive, purple—no consensus, and light gray—no literature), while the texture of the line indicates the number of studies identified (dashed represents two or fewer studies; solid indicates >2)

In order to assess how much published research supported the stressor pathways identified in the expert‐based conceptual model, we performed a keyword‐focused literature review. We searched Web of Science (www.webofknowledge.com) and targeted the driver‐to‐pressure pathways and the pressure‐to‐kelp response pathways. We originally focused on topics for bull kelp canopy but our literature review was expanded to include all kelps since it was the general consensus that pathways would be similar for other species. While some driver‐to‐pressure pathways were mediated via complex pathways not necessarily identified by the expert panel, we focused the literature search strings on the main drivers and pressures (Figure 4). Search strings were created based on descriptions of each driver used in the focus group so that a driver like *upland development*, for example, included logging, agriculture, urban areas, industrial activity, and dams (Appendix S1—all search strings**)**. The geographic scope of literature searches included the majority of areas where kelps grow, excluding mesophotic populations and high‐latitude regions that experience seasonal ice coverage. We excluded reviews and meta‐analyses to prevent double‐counting of empirical experiments. While the focus group primarily assessed pressures on bull kelp mature sporophytes, our literature search included any kelp species and life‐history stage. We also included known foundational papers that did not appear in Web of Science due to the age of the paper. Considering that Web of Science coverage of papers published prior to the 1990s is incomplete, we may have missed other relevant studies that were not previously known to the authors.

The findings from relevant studies were summarized to aid in comparisons across studies. Location was split into the Salish Sea or the broader temperate Pacific coast, which included studies from the low‐latitude range limit of kelp to the high‐latitude limit of sea ice formation. The directionality of the relationship between the driver and pressure or between the pressure and kelp response was categorized as positive, negative, neutral (i.e., no relationship), or other (e.g., synergistic, antagonistic, threshold effect). Research methods included observational, experimental, and modeling. For the pressure‐to‐kelp response pathways, we also recorded the kelp species, guild (e.g., floating or nonfloating), and life‐history stage (e.g., spore, gametophyte, juvenile sporophyte, sporophyte). Publications were counted multiple times if they contributed to multiple linkages (e.g., multiple locations, focal species, drivers, or pressures), resulting in a total study count that exceeded the final number of publications. Due to the high consensus that climate change and sea surface temperature are positively related (IPCC, 2019), we did not search the global literature for the climate change‐sea surface temperature Driver–Pressure pathway.

## 3 RESULTS

The expert focus group identified six primary Drivers (indirect stressors from human activities) and 10 primary Pressures (direct physical and ecological stressors on kelp) with four intermediate states (Figure 3). This resulted in a total of 51 pathways: 36 pathways between Drivers and Pressures, including intermediate states, 6 pathways representing how Pressures can impact other Pressures, and 9 pathways between Pressures and kelp State. The literature review that we performed was based on a slightly simplified diagram that did not include intermediate states and therefore focused on a total of 45 pathways: 30 Driver to Pressure pathways, 6 Pressure to Pressure pathways, and 9 Pressure to kelp State pathways (Figure 4). We identified 767 studies that tested the relationship of the identified pathways, 57 of which were from the Salish Sea. We found literature to represent all identified pathways among the global studies, but we only found literature representing the Salish Sea for 21 of the 45 pathways.

### 3.1 Human impacts on the environment (Drivers–Pressures)

The Drivers identified to be influencing the most Pressures were vessel traffic (7 pathways; 22 studies), climate change (6 pathways; 40 studies), and upland development (6 pathways; 61 studies). Pressures that were influenced by the most Drivers or other Pressures included water clarity (6 pathways; 48 studies) and benthic sedimentation (5 pathways; 45 studies). Where literature was available from both the Salish Sea and other temperate regions, the direction of the relationship between a given Driver and Pressure was often the same. However, there were a few notable exceptions: climate change and nutrients; human alterations to trophic structures and epiphytes or kelp tissue damage; shoreline or upland development and benthic sedimentation; nutrients and epiphytes; and upland development and water clarity. Of all Driver–Pressure pathways investigated, we found the fewest studies that represented impacts of vessel traffic (22 studies, none from the Salish Sea) and nutrients (22 studies, 4 from the Salish Sea), while the impacts of climate change and dredging were the most represented (respectively: 40 papers, 10 from the Salish Sea; and 67 papers, 1 from the Salish Sea) (Figure 4).

#### 3.1.1 Shoreline development

This driver encompassed shoreline hardening and over‐ and near‐water structures such as docks. The experts identified it as impacting salinity, nutrient levels, temperature, water clarity, and benthic sedimentation (Figure 3). Among the global literature, shoreline development was associated with higher substrate and air temperatures in the intertidal zone and increased nearshore nutrient levels, especially when it was also associated with significant upland development (Jordan et al., 2018). Impacts on water clarity and sedimentation were more variable and depended on local current patterns, geology, and the specific type of development. Shoreline development generally had a neutral relationship with salinity. Among the Salish Sea literature, shoreline development was associated with an increase in the size and number of cobbles, interpreted here as a decrease in benthic sedimentation (Dethier et al., 2016). Unlike in the global literature, we found no literature from the Salish Sea on impacts of shoreline development on salinity, nutrients, or water clarity (Figure 4).

#### 3.1.2 Climate change

The focus group indicated that climate change would impact salinity, nutrients, temperature, water clarity, benthic sedimentation, and contaminant levels (Figure 3). Some pathways, such as the relationship between climate change and water temperature, had very high consensus among studies and are well established in the global literature (IPCC, 2019), while others, such as the relationship with salinity or nutrient levels, were more variable and place‐dependent. There was generally a positive relationship in the literature between precipitation and nearshore salinity and terrestrially derived nutrients, contaminants, and sediments, though the relationship between climate change and precipitation was location‐dependent (Dwight et al., 2011; Vuorinen et al., 2015; Wikner & Andersson, 2012). Climate change can also result in decreased nutrient concentrations due to increased stratification reducing upwelling intensity of nutrient‐rich deep water (Holt et al., 2016; Kamykowski & Zentara, 2005; Law et al., 2018), decreased water clarity due to increased primary production (Capuzzo et al., 2018), and increased concentrations of suspended sediments from increased storm frequency and melting tidewater glaciers (Carney & Edwards, 2010, Suursaar et al., 2011). The Salish Sea is a region of high precipitation that will likely increase given climate change (Mote & Salathé, 2010). For example, the timing of the Fraser River spring outflow is trending earlier, causing salinity decreases earlier in the season (Riche et al., 2014). The relationship with nutrients was less clear in the Salish Sea where nutrients are delivered via upwelling, which is projected to decrease, and terrestrial runoff, which is generally increasing. We found no research on climate change impacts on sedimentation or contaminants in the Salish Sea (Figure 4).

#### 3.1.3 Upland development

This category encompassed land‐use changes to watersheds, including logging, agriculture, urbanization, dams, and industrial activities. The experts identified impacts on salinity, nutrients, temperature, water clarity, sedimentation, and contaminants (Figure 3). In the literature, upland development had a negative relationship with salinity (Corcoran et al., 2010); a strong positive relationship with nutrients due to industrialized agriculture, urban wastewater, atmospheric deposition, and fish processing plants (Canton et al., 2012; Garrido‐Pérez et al., 2002; Kim et al., 2015; Lalonde & Ernst, 2012); and a strong negative relationship with water clarity (Desmond et al., 2015). Upland development also had a positive relationship with benthic sedimentation driven by land clearing, mining, poorly handled wastewater, and coal‐fired power plant emissions (González et al., 2014; Gorostiaga & Díez, 1996); and a strong positive relationship with nearshore contaminants, especially heavy metals and petrochemicals due to current and historical military, industrial, residential, and agricultural effluent (Harris et al., 2011; O’Connor, 2002; Xu et al., 2016). One study on the impacts of development on nearshore sea surface temperature found an urban heat island effect in an adjacent bay (Jung, 2008). We found 14 studies from the Salish Sea documenting strong positive relationships between upland development and contamination due to present and historic military and industrial activity and vehicle exhaust (Long et al., 2005; Martin & Nesbitt, 2015; Poirier, 2006). We also found a positive relationship between upland development and water clarity, focusing on water clarity decreases after the removal of the Elwha Dam (Glover et al., 2019), a negative relationship with benthic sedimentation, also focusing on the Elwha Dam removal (Glover et al., 2019; Rubin et al., 2017) and a mixed relationship with nutrient levels (Mackas & Harrison, 1997). We interpreted dam removal as a decrease in upland development. We identified no studies from the Salish Sea on upland development impacts on salinity or temperature (Figure 4).

#### 3.1.4 Vessel traffic

Vessel traffic, which included large and small vessels, was suggested to impact nutrients, temperature, water clarity, contaminants, and incur potential mechanical damage to kelps (Figure 3). While there was strong support in the global literature for vessel traffic increasing nearshore contaminants and decreasing water clarity (Bowman et al., 2003; Choi et al., 2009; Garel et al., 2008), the other pathways had little research in the global literature and no research in the Salish Sea (Figure 4). Given the increases in recreational and commercial vessel traffic in the nearshore environment, this is an area that deserves more investigation.

#### 3.1.5 Dredging

The focus group determined that dredging impacted water clarity, sedimentation, and contaminants (Figure 3), all of which had strong support in the global literature. These included a negative relationship with water clarity and positive relationship with sedimentation and contaminants (de Jonge et al., 2014; Hedge et al., 2009; Nielsen et al., 2015). We found few published studies from the Salish Sea for these pathways, despite dredging being a common practice in ports across the region (Spadaro et al., 1993, NMFS, 2015) (Figure 4).

#### 3.1.6 Human impacts to trophic structures

This category included the effects of fishing and hunting, invasive species introductions, and the reintroductions of previously extirpated species. The experts identified it as influencing mechanical damage of kelp via grazing, algal competition with kelp, and epiphytic growth on kelp (Figure 3). While the broader coast literature had stronger support for positive relationships between human impacts and mechanical damage, algal competition, and epiphytes, the limited literature from the Salish Sea indicated the opposite (Figure 4). One study used heron exclusions to mimic human‐induced declines in wading bird populations and found this resulted in decreases in epiphytes on eelgrass (Huang et al., 2015) while another experimentally removed urchins to simulate otter reintroductions, which resulted in no change to the macroalgal community (Carter et al., 2007). This topic deserves greater attention considering that many present‐day fisheries target high‐trophic level fish species and historic fisheries heavily targeted marine mammals. These alterations to trophic structures in the past and present may be releasing herbivores from predation pressure resulting in an increase in grazing pressure (Dunn et al., 2017).

#### 3.1.7 Temperature

The focus group identified temperature as impacting rates of damage of kelp tissues, algal competition with kelp, and epiphytic growth on kelp (Figure 3). We found many studies on the relationship between temperature and damage due to grazing, with extremely variable relationships because grazer response to temperatures was temperature‐specific (minor increases in temperature may increase activity but activity will then decline as temperature continues to increase due to higher mortality rates; for example, Cardoso et al., 2017; Miranda et al., 2019), species‐specific (temperature thresholds vary by species; e.g., Legrand et al., 2017), seasonally specific (activity will more likely increase during winter months than summer months with elevated temperatures; Werner et al., 2016), and exposure‐dependent (shorter‐term exposure to elevated temperatures will more likely decrease activity rates; Russell et al., 2013). The pathways for algal competition and epiphytic growth had limited studies that indicated positive relationships with temperature (Smale et al., 2015; Werner et al., 2016). We found no research on these pathways from the Salish Sea (Figure 4).

#### 3.1.8 Nutrients

Nutrients were identified to impact water clarity, marine macrophyte tissue damage, algal competition with kelp, and epiphytic growth on marine macrophytes (Figure 3). We found strong support in the literature for a positive relationship between nutrients and algal competition in which higher nutrient loads allow fast‐growing species such as *Ulva* sp. to outgrow kelp species (Pederson & Borum, 1997). The relationship with epiphytic growth was less clear, with some epiphytic species benefiting from higher nutrient concentrations, while others were unaffected (Karez et al., 2004). We found support for both relationships in the Salish Sea literature, with research focusing on seagrass systems (Nelson et al., 2008; Nelson & Waaland, 1997). Among the global literature, nutrients were shown to have a negative relationship with water clarity (Boesch, 2000) and a positive relationship with tissue damage for macrophyte species, though this was mediated by epiphytic growth or grazing rates (Ruesink, 2016; Tegner et al., 1995). No studies were found for the Salish Sea on water clarity or tissue damage (Figure 4).

### 3.2 Environmental impacts on kelp (Pressures–State)

**FIGURE 5:**
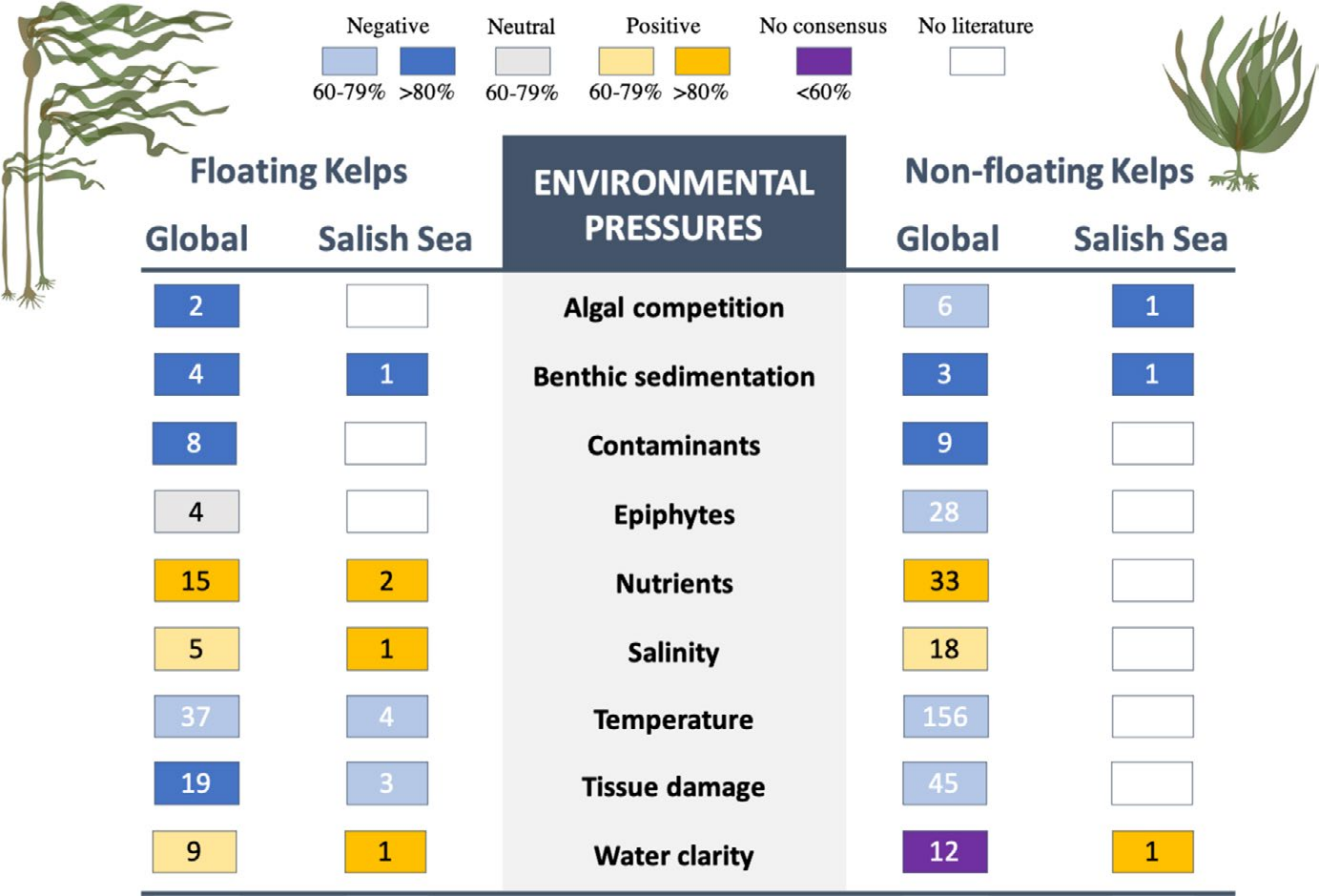
Results of literature searches of the Pressures impacting floating and nonfloating kelp species in the Salish Sea and temperate coasts wherever kelps are found. The numbers in each box represent the number of studies identified (no number indicates a pathway for which no studies were identified). The color of each box represents the direction of the relationship (blue represents negative, gray—neutral, orange—positive, purple—no consensus, and white—no literature). Shading of each color represents the degree of consensus among the studies identified in the direction of the relationship, with darker shades representing high consensus (>80%) and lighter shades representing medium consensus (60%–79%). Below 60% was categorized as no consensus

The focus group identified 10 pressures facing kelp in the Salish Sea, including salinity, nutrients, temperature, water clarity, benthic sedimentation, contaminants, mechanical damage, grazing, algal competition, and epiphytes (Figure 3). We found a total of 430 studies representing these Pressure to State pathways with the most literature on the effects of temperature (197 studies, 4 from the Salish Sea) and the least on benthic sedimentation (7 papers, 1 from the Salish Sea). The literature review revealed a general consensus in the direction of the relationships between a given pressure and a kelp state when compared between Salish Sea and global temperate literature, between floating and nonfloating kelp species, and among kelp life‐history stages; however, many research gaps remain for kelp populations in the Salish Sea and for kelp early life stages (Figure 5). The vast majority of literature investigated the sporophyte stage (370 studies) (Figure 2b).

#### 3.2.1 Salinity

Salinity has a strong influence on the distribution and growth of both understory and canopy kelps. The only paper documenting the impacts of salinity on Salish Sea kelp populations investigated the influence of salinity on the distribution of *Macrocystis pyrifera* found only near the Strait of Juan de Fuca western entrance, where salinity is equivalent to that of the open Pacific Ocean. Experimental transplants demonstrated that this pattern is driven by environmental sensitivity to reduced salinity or to the interacting effects of reduced salinity and increased summertime temperatures (Druehl & Hsiao, 1977). Similar patterns of reduced growth and health under reduced salinity conditions have been documented elsewhere in the distribution of *M*. *pyrifera* (Rodríguez et al., 2019). While only one identified study looked directly at the impacts of salinity on kelps in the Salish Sea, similar negative impacts of hyposalinity (i.e., reduced salinity relative to ambient) have been documented in other temperate systems on some species also found in the Salish Sea, such as *Saccharina latissima* and *Nereocystis luetkeana*. Under experimental settings, reduced salinity can lower growth rates (Li et al., 2020), cause blistering or bleaching (Vettori et al., 2020), induce a physiological stress response (Bollen et al., 2016; Li et al., 2020; Monteiro et al., 2019; Mortensen, 2017), and limit recruitment (Rodríguez et al., 2019). This has been documented in multiple species of both canopy (e.g., *Macrocystis pyrifera*) and understory kelps (e.g., *Saccharina latissima*, *Laminaria* spp.). Very few studies have looked at the effects of increased salinity relative to those naturally experienced by kelp, likely because hypersaline environments are not as common in temperate waters. However, kelps may also be sensitive to increased salinity (e.g., 50 ppm; Nitschke & Stengel, 2014), suggesting that there is an optimal salinity at which kelps can grow. Salinity can also alter the composition of kelp surface microbiomes, with lower salinity driving reduced microbial abundance and diversity (Weigel & Pfister, 2019). However, the physiological and/or ecological impacts of microbial diversity remain unclear.

#### 3.2.2 Nutrients

The positive relationship between nutrient concentrations, especially nitrogen, and kelp growth is well established in the global literature, though extremely high nutrient loadings can result in kelps being outcompeted by fast‐growing turf species (see *Algal competition* below). We found two studies from the Salish Sea that addressed the relationship between nutrient concentrations and kelp. One study experimentally added nutrients in the intertidal zone at the western entrance of the Strait of Juan de Fuca and observed no increase in kelp growth, indicating that these kelps were not nutrient‐limited (Pfister & Alstyne, 2003). The other used long‐term data on *N*. *luetkeana* canopy extent and water column nitrogen concentrations in the South Puget Sound region to reveal a robust positive relationship between nutrient levels and canopy persistence (Berry et al., 2021) (Figure 5).

#### 3.2.3 Temperature

Kelps have an optimum temperature range that differs across species and potentially populations. Most studies from the global literature focused on kelp performance when exposed to higher temperatures, resulting in a generally negative relationship between temperature and kelp performance in our literature reviews; however, extremely low temperature can also have negative impacts on kelp growth. We identified four studies from the Salish Sea, two of which documented declines in *N*. *luetkeana* or *M*. *pyrifera* canopies during years of warm sea surface temperatures (Pfister et al., 2018; Schroeder et al., 2020), one showed long‐term contractions of *N*. *luetkeana* canopies related to long‐term increases in sea surface temperature (Berry et al., 2021), and one experimental study that demonstrated highest growth of *N*. *luetkeana* between 12°C and 14°C, indicating a nonlinear relationship between growth and temperature (Supratya et al., 2020) (Figure 5).

#### 3.2.4 Water clarity

Both floating and nonfloating species tended to have positive relationships with increasing water clarity, though data were limited for the Salish Sea, and experiments and observations from the broader temperate coast indicated variability in this relationship (Figure 5). In the Salish Sea, we only encountered one study that found dramatic decreases in the cover of floating and nonfloating kelp species after the removal of a large dam released tons of sediment into the nearshore areas of the Strait of Juan de Fuca, resulting in high turbidity and decreased light (Rubin et al., 2017). Kelp populations showed some recovery as the influx of sediment slowed and the water clarity improved (Rubin et al., 2017). Most of the studies from outside of the Salish Sea found a positive relationship between water clarity and kelp performance in both floating and nonfloating species. The next most common finding was a nonlinear relationship, in which the negative impact of reduced light was reduced (antagonistic effect) given high nutrient levels (Buschmann et al., 2014) and local adaptation or acclimation (Gerard, 1990), or kelps responded poorly to both too much light and too little light given high temperatures, indicating both a threshold effect of high light and a synergism between temperature and light stress (Mabin et al., 2019). The only study that found a negative relationship between light and kelp performance focused on the understory species *Laminaria pallida* across a natural turbidity gradient in South Africa and Namibia. The study found that this species became increasingly dominant as turbidity increased, likely because it was more resistant to low‐light conditions than *Ecklonia maxima*, a floating canopy species (Rothman et al., 2017). One study observed morphological changes in *E*. *radiata* in more turbid sites, suggesting phenotypic responses that are better suited to low‐light conditions (Blain et al., 2020).

#### 3.2.5 Benthic sedimentation

All studies reviewed found a strong negative relationship between benthic sediment accumulation and kelp survival with near total extirpation of floating and nonfloating kelp species after the introduction of large volumes of sediment from mine tailings (González et al., 2014); landslides (Schiel et al., 2019); discharged sewage effluent (Stull, 1996); and in the Salish Sea, dam removal (Rubin et al., 2017) (Figure 5).

#### 3.2.6 Contaminants

Contaminants, including heavy metals, sewage, and petrochemicals, reduced kelp performance in all studies except one, in which reduced herbivory in a polluted port resulted in increased *E*. *radiata* cover (Fowles et al., 2018). These findings were consistent across kelp guilds, though notably we found no studies on the relationship between contaminants and kelp in the Salish Sea (Figure 5).

#### 3.2.7 Mechanical damage

Tissue damage from biological (e.g., grazers) and hydrological (e.g., waves and currents) and mechanical forces play an important role in structuring kelp forest dynamics. The available literature reflects this, describing losses to kelp abundance and biomass following large storm and/or wave events (Castorani et al., 2018; Filbee‐Dexter & Scheibling, 2012), or major population increases of herbivores such as urchins (Morris & Blamey, 2018; Norderhaug et al., 2020). Other factors, such as kelp entanglement and abrasions or cuts compound storm and wave disturbance and contribute to increased kelp mortality (Burnett & Koehl, 2018; DeWreede et al., 1992). While it is well documented in the global literature that tissue damage from grazing or water movement has a negative impact on kelp growth and survival, we found only three studies in the Salish Sea (Figure 5). In the turbulent currents of the San Juan Islands in the Salish Sea, minor physical damage to *Nereocystis luetkeana* stipes by the herbivorous snail *Lacuna vincta* can increase mortality in areas of high tidal currents (Duggins et al., 2001), and mechanical damage caused by kelp crabs can reduce *N*. *luetkeana* growth as crabs showed a strong preference for *N*. *luetkeana* over *M*. *pyrifera* (Dobkowski, 2017).

#### 3.2.8 Algal competition

Kelp forests are characterized by frequent disturbance making algal competition, in the form of succession, a fact of life in these habitats. In the literature reviewed, we found primarily negative relationships between competition and kelp State, driven by succession after disturbance (Yoneda et al., 2007), invasion by the green algae *Codium fragile* (Levin et al., 2002), and extensive shading or lack of available space due to thick understory algae (Hernández‐Carmona et al., 2000; Tatsumi & Wright, 2016). However, *Saccharina sessilis* recruitment improved in the presence of other understory algal species (Barner et al., 2016). In the Salish Sea, we found one study that found fewer native kelp *Laminaria bongardiana* in plots with the invasive brown algae *Sargassum muticum* (Britton‐Simmons, 2004) (Figure 5).

#### 3.2.9 Epiphytes

Epiphytes are generally benign in areas where epiphytes and kelps have co‐evolved. However, in areas where an epiphytic species has been introduced, such as the bryozoan *Membranipora membranacea* in the Northwest Atlantic, or changing oceanographic patterns alter interactions, such as *M*. *membranacea* in the Northeast Pacific, epiphytes negatively affect kelp populations by overgrowing fronds and preventing photosynthesis, reducing flexibility and causing breakage, or resulting in blade mortality (Saunders & Metaxas, 2008). We found no literature on epiphyte/kelp interactions from the Salish Sea (Figure 5).

## 4 DISCUSSION

In management scenarios where data are limited, it is common to elicit the advice and opinions of regional experts to provide the best available science for the management decision‐making process, particularly when related to questions concerning how ecosystems or habitats may respond to natural and anthropogenic pressures (Donlan et al., 2010; Martin et al., 2012; Ryder et al., 2010; Turner, 2010). Here, we invited researchers and resource managers to develop an inclusive conceptual model of the pressures, and ultimately human activities, that affect the status and trends (State) of kelp in the Salish Sea. This work was motivated by disturbing disappearances of bull kelp forests in Puget Sound, and the paucity of local quantitative information to explain this decline (Berry et al., 2021). Losses of these and other kelp forests in the Salish Sea could negatively impact the availability of nearshore habitat to commercially and ecologically significant species (Teagle et al., 2017), while also reducing the productivity of nearshore environments (Duggins et al., 1989). Consequently, management actions that facilitate the recovery and conservation of kelp forests in the Salish Sea would increase the provisioning of ecosystem services and ensure the long‐term functioning and productivity of coastal ecosystems. It is important to note that another set of individuals from a greater diversity of the general public, including individual citizens, local stakeholders, and more representation from tribes and First Nations may have developed different models (Reid et al., 2020; Ressurreição et al., 2012; Rosellon‐Druker et al., 2019; Stier et al., 2017). The combination of this focus group's conceptual model and the relatively consistent support for these pathways found in the literature review suggests expert perceptions of the system are a good starting point for understanding the dynamics important to informing the decision‐making process for conservation and management of kelp in the Salish Sea. The validation of this conceptual model, in addition to quantifying the strength of directionality in relationships, may provide the foundation for predicting anthropogenic impacts on kelp forests in the Salish Sea using semiquantitative and quantitative modeling techniques that could give further insight into the relative importance of each linkage on kelp forest persistence (Hollarsmith et al., 2021). However, further inclusion of regional stakeholders and the general public in participatory processes related to this conceptual model and specific management actions will ensure other nodes of the social–ecological system are accounted for in the decision‐making process (Dietz, 2013; Stier et al., 2017).

Overall, we found considerable support in the literature for a majority of the Driver–Pressure–State pathways identified in the conceptual model developed by the expert‐based focus group. However, the vast majority of supporting studies were based on research performed outside of the Salish Sea region, 87% for Driver‐to‐Pressure pathways and 96% for Pressure‐to‐State pathways. The Salish Sea is an oceanographically diverse and complex set of inland waterways with estuarine‐style circulation patterns that leads to net seaward flow of brackish surface layers and net landward flow of deep, dense oceanic waters (Alford & MacCready, 2014; Babson et al., 2006; Masson, 2002). There are numerous sills that constrict and alter geomorphological and oceanographic processes that isolate specific regions at various temporal and spatial scales. These characteristics may impose environmental conditions for kelp that are dissimilar from other coastal kelp habitats where much of our mechanistic understanding of these Driver–Pressure–State relationships have been studied. The lack of data to support these relationships directly may limit the specificity of advice for the conservation and management of kelp in this region. Notably, however, we found generally high consensus in directional relationships between the Salish Sea and global literature, so the results from the global literature may be a good approximation of processes in the Salish Sea.

While we found multiple studies to support the impacts of expert‐identified Pressures on various kelp species, these studies were not evenly distributed across the stages that comprise the complex life cycle of kelp. The vast majority of studies focused on the adult sporophyte stage of kelp, which is the stage that provides the most three‐dimensional habitat structure and organic carbon to the kelp forest ecosystem. However, the earlier microscopic life stages may be an important and largely invisible bottleneck in the kelp reproductive cycle (Hollarsmith et al., 2020; Muth et al., 2019). For pathways that had studies on multiple life‐history stages, there was a high degree of consensus about the direction of the impact, with the exception of water clarity, which was largely positively related to sporophyte performance metrics. The only study investigating other life stages found that for populations from turbid areas, water clarity did not impact gametophytes (Gerard, 1990). Generally, this suggests that results for one life‐history stage may be able to be cautiously extrapolated to other stages; however, more research on environmental impacts to spore, gametophyte, and microscopic sporophyte stages is warranted.

This literature review was designed to evaluate the pathways identified in the focus group's conceptual model, not to seek out any missing pathways; however, during our keyword searches, we did identify four driver‐to‐pressure pathways and two pressure‐to‐kelp pathways that did not fit into the expert‐identified pathways and that may need to be considered going forward. First, the human activity of net‐pen aquaculture was identified in the broader temperate coast literature searches as increasing nearshore contamination, benthic sedimentation, and nutrients; and decreasing water clarity (Claudet & Fraschetti, 2010; Feng et al., 2004; Lalonde & Ernst, 2012; Wang et al., 2020). Second, invasive algal species, included under the category of human impacts to trophic structures, may enhance benthic sedimentation rates (Bulleri et al., 2010). Third, temperature has been found to be positively related to epiphyte growth (Werner et al., 2016); and fourth, shoreline development can alter nearshore substrate (Dethier et al., 2016). We also found that viral disease can negatively impact kelp growth and survival (Beattie et al., 2018), which may not be currently affecting kelp in this region but could represent a future threat, considering viral outbreaks have recently affected other taxa in the region (Hewson et al., 2014). Another omission was the direct impact of water motion, currents, and wave action on kelp performance, which was included as a mediator between drivers and pressures in the initial diagram. We encountered evidence that suggests it has a direct impact on kelp (Berry et al., 2021; Kregting et al., 2016; Millar et al., 2020; Peteiro & Freire, 2013; Starko et al., 2019). There are likely other direct or indirect pathways not identified by the expert‐based conceptual model that the literature search also did not capture as the perceptions, knowledge, and biases of experts can vary widely, even within the narrow demographic range of ‘kelp experts’ used in this study (Drescher et al., 2013; Martin et al., 2012; Stier et al., 2017).

### 4.1 Research priorities for the Salish Sea

While studies from the global literature may serve as effective approximations of processes in the Salish Sea, the extreme paucity of literature on pressures impacting floating and nonfloating kelp species in the region indicates an urgent need for research to inform local resource management decisions for kelp conservation and recovery. Situated in a temperate rainforest and composed of deep fjords and large glacial‐fed estuaries, the oceanography of the Salish Sea is distinct from many of the other regions represented in our global temperate literature search. The estuarine environment is unusual for kelp, with periodic or seasonal changes in salinity, temperature, turbidity, and other water column parameters that are often much larger than observed in open coast environments where most kelps are found (MacCready et al., 2021). Research has shown that kelps can exhibit population‐level differences in response to environmental stress (Buschmann et al., 2004; Flukes et al., 2015; Hollarsmith et al., 2020; King et al., 2019), and recent population genetic work on bull kelp in the Salish Sea revealed distinct genetic clusters that aligned with oceanographic currents, geographic and benthic features, and environmental variables (Gierke, 2019). Evidence for genetic structure further supports the need for more research on Salish Sea kelp populations to more accurately understand current and future changes in kelp extent across the different basins.

Human actions that are managed at the local level, such as nearshore and upland development and regional fisheries, are some of the Drivers that most need research in the Salish Sea to support management decision making. Historic fisheries and other human activities in the Salish Sea region depleted a number of species, including Pacific cod (*Gadus macrocephalus*), Pacific hake (*Merluccius productus*), rockfish (*Sebastes* spp.), and walleye pollock (*G*. *chalcogrammus*) (Essington et al., 2021; Gustafson et al., 2000; Harvey et al., 2012; Palsson et al., 2009; Williams et al., 2010). Of note, rockfish populations have declined by an estimated 70% over the past 40 years (Drake et al., 2010; Tolimieri et al., 2017). In the same time period, pinniped populations have increased dramatically after the passage of the Marine Mammal Protection Act in 1972 (Jeffries et al., 2003; Johannessen & McCarter, 2010). These species, among others, occupy mid‐ to top‐trophic levels, and they likely play an important role in the Salish Sea ecosystem by maintaining healthy linkages with its trophic systems. For instance, various rockfish species have been found to feed on kelp crabs and other invertebrates that eat kelp in Puget Sound (Washington et al., 1978). The decline of rockfish and other fish that eat or impact grazer populations may be contributing to the decline of kelp (Calloway et al., 2020). However, we found very limited literature regarding trophic changes impacting kelp within the study area, indicating a large gap in the primary literature. Given the ubiquity of the trophic cascade impacts to kelp worldwide, it is likely this dearth of research represents a data gap for the region and would be worth further investigation.

Similarly, research of the more potentially acute conditions in the Salish Sea related to human activity, such as contaminants, impacts of vessel traffic, water quality changes, and nearshore and upland development are warranted. Watersheds that drain into the Salish Sea are extensively logged (Hansen et al., 2013), human populations in the region are increasing rapidly (OFM, 2020), and the timing and magnitude of delivery of fresh water is changing as climate change results in more rain than snow and glaciers rapidly recede (Mote & Salathé, 2010; Riedel & Larrabee, 2011). At the same time, stronger environmental protection legislation has improved water and air quality and reduced historic contaminant and pollutant levels, though emerging pollutants remain a concern (EPA, 2021). Despite these substantial changes to hydrology and environmental quality in the region, we found very few studies that explicitly address how these changes impact the marine environment. Of note, research of these factors in the Salish Sea should account for the regional diversity of environmental conditions that naturally affect water retention times, temperature regimes, and consequences of changing contaminant identities, concentrations, and distributions throughout the region.

## 5 CONCLUSION

Our use of expert opinion and a structured literature review resulted in a comprehensive framework to support management decision‐making despite a paucity of local data. Ultimately, management outcomes will depend on a number of external factors but by utilizing multiple, informed lines of evidence to inform management decision making one greatly increases the chances of a positive outcome. The complexities of modern anthropogenic stressors on nearshore environments require a diverse suite of approaches to identify relevant pathways and to prioritize knowledge gaps for additional quantitative research. By gathering a focus group of relevant experts on the Salish Sea, we were able to rapidly diagram the multiple stressor pathways that are likely contributing to regional kelp decline and use this diagram to inform a systematic literature survey that was then used to identify critical knowledge gaps to direct future research efforts. This targeted, multistage approach allowed us to resolve complex linkages that otherwise would have been missed by using only a single approach. The results inform future research directions while also providing a tool managers can use in the absence of regional quantitative data. Kelps provide important habitat in the Salish Sea, and the loss of this habitat will likely have cascading impacts on other fish, invertebrate, and mammal species that are part of nearshore food webs and the humans that rely upon them. The approach developed here can be extended to other ecosystem‐based management decision‐making processes where quantitative data are lacking, and expert opinion can be incorporated in a more standardized way by linking directly to a conceptual model of the system. Managing and restoring threatened ecosystems such as the Salish Sea, which are under increasing pressure from both the influences of climate change and human intervention, will require us to draw upon both qualitative and quantitative data and expert opinions from many different sources in order to best manage these complex and dynamic ecosystems.

## CONFLICT OF INTEREST

The authors have no competing interests to declare.

## AUTHOR CONTRIBUTIONS

**Jordan A. Hollarsmith:** Conceptualization (lead); Data curation (lead); Investigation (equal); Methodology (equal); Project administration (lead); Visualization (equal); Writing – original draft (lead); Writing – review & editing (equal). **Kelly Andrews:** Conceptualization (equal); Data curation (equal); Investigation (equal); Methodology (equal); Visualization (equal); Writing – original draft (equal); Writing – review & editing (equal). **Nicole Naar:** Conceptualization (equal); Data curation (equal); Investigation (equal); Methodology (equal); Project administration (equal); Visualization (equal); Writing – original draft (equal); Writing – review & editing (equal). **Samuel Starko:** Conceptualization (equal); Data curation (equal); Investigation (equal); Methodology (equal); Project administration (equal); Writing – original draft (equal); Writing – review & editing (equal). **Max Calloway:** Conceptualization (equal); Investigation (equal); Methodology (equal); Visualization (equal); Writing – original draft (equal); Writing – review & editing (equal). **Adam Obaza:** Conceptualization (equal); Investigation (equal); Methodology (equal); Visualization (equal); Writing – original draft (equal); Writing – review & editing (equal). **Emily Buckner:** Conceptualization (equal); Investigation (equal); Methodology (equal); Visualization (equal); Writing – original draft (equal); Writing – review & editing (equal). **Daniel Tonnes:** Conceptualization (equal); Investigation (equal); Methodology (equal); Project administration (equal); Visualization (equal); Writing – review & editing (equal). **James Selleck:** Conceptualization (equal); Methodology (equal); Writing – review & editing (equal). **Thomas W. Therriault:** Conceptualization (equal); Investigation (equal); Methodology (equal); Writing – original draft (equal).

## Supporting information

Appendix S1

## Data Availability

All data used in this study, namely the literature that contributed to the literature review, can be found in the Appendix S1.
