# Supplementary material for: Toward a conceptual framework for managing and conserving marine habitats: A case study of kelp forests in the Salish Sea
Source: Ecol Evol. 2022 Jan 12;12(1):e8510. doi: 10.1002/ece3.8510 (PMC8809449; doi:10.1002/ece3.8510)
Supplement: Supplementary file 1 — Appendix S1 [file ECE3-12-e8510-s001.zip › ece38510-sup-0003-AppendixS1.docx]

**Appendix 1: Search strings**

All searches were completed by December 2020.

Drivers

Human impacts to trophic structures and epiphytes:

- ((invasive OR reintroduc* OR fisher* OR fishing) AND epiphyt* AND marine NOT coral NOT tropic*)

Human impacts to trophic structures and algal competition:

- (invasive OR reintroduc* OR fisher* OR fishing) AND algal competition NOT coral NOT tropic*

Nutrients and epiphytes

- "nutrient* AND epiphyte* AND ("Salish Sea" OR "Puget Sound" OR "Strait of Georgia" OR "San Juan Islands" OR "Gulf Islands" OR "Vancouver Island" OR "Washington" OR "British Columbia") NOT terrestrial*
- "nutrient* AND epiphyte* AND marine* NOT tropic* (category = ''marine freshwater biology')

Nutrients and algal competition:

- ("nutrient* AND algal competition AND ("Salish Sea" OR "Puget Sound" OR "Strait of Georgia" OR "San Juan Islands" OR "Gulf Islands" OR "Vancouver Island" OR "Washington" OR "British Columbia"))
- "nutrient* AND epiphyte* AND marine* NOT tropic* (category = ''marine freshwater biology')

Nutrients and water clarity

- ʺnutrient* AND (turbid* OR "water clarity" OR light availab*) AND (ʺSalish Seaʺ OR ʺPuget Soundʺ OR ʺStrait of Georgiaʺ OR ʺSan Juan Islandsʺ OR ʺGulf Islandsʺ OR ʺVancouver Islandʺ OR ʺWashingtonʺ OR ʺBritish Columbiaʺ)

Temperature and algal competition

- temperature* AND algal competition AND marine* NOT tropic*

Temperature and epiphytes

- "temperature* AND epiphyte* AND marine* NOT tropic*

Temperature and grazing

- "temperature* AND grazer* AND marine* NOT tropic*

Dredging and contaminants

- (dredg* AND marine AND (pollut* OR contamin* OR "water quality"))

Dredging and benthic sedimentation

- dredg* AND (benth* AND sediment* AND marine)

Dredging and water clarity

- dredg* AND (turbid* OR "water clarity" OR light availab*)

Upland development and contaminants

- (logging OR agricultur* OR urban* OR dam* OR industr*) AND (pollut* OR contamin*) AND marine AND ("Salish Sea" OR "Puget Sound" OR "Strait of Georgia" OR "San Juan Islands" OR "Gulf Islands" OR "Vancouver Island" OR "Washington" OR "British Columbia")

Upland development and nutrients

- (logging OR agricultur* OR urban* OR dam* OR industr*) AND marine AND nutrient* NOT tropic*
- (logging OR agricultur* OR urban* OR dam* OR industr*) AND nutrient* AND ("Salish Sea" OR "Puget Sound" OR "Strait of Georgia" OR "San Juan Islands" OR "Gulf Islands" OR "Vancouver Island" OR "Washington" OR "British Columbia")

Upland development and salinity

- (logging OR agricultur* OR urban* OR dam* OR industr*) AND salin* AND nearshore AND marine NOT tropic*

Upland development and benthic sedimentation

- (logging OR agricultur* OR urban* OR dam* OR industr*) AND benthic AND sediment* AND marine AND ("Salish Sea" OR "Puget Sound" OR "Strait of Georgia" OR "San Juan Islands" OR "Gulf Islands" OR "Vancouver Island" OR "Washington" OR "British Columbia")

Upland development and temperature

- (logging OR agricultur* OR urbaniz* OR industr*) AND marine AND temperature NOT tropic* NOT *arctic)

Upland development and water clarity

- (logging OR agricultur* OR urban* OR dam* OR industr*) AND (turbid* OR "water clarity" OR light availab*) AND marine AND ("Salish Sea" OR "Puget Sound" OR "Strait of Georgia" OR "San Juan Islands" OR "Gulf Islands" OR "Vancouver Island" OR "Washington" OR "British Columbia")
- ((logging OR agricultur* OR urban* OR dam* OR industr*) AND (turbid* OR "water clarity" OR "light availability") AND marine NOT tropic*)

Vessel traffic and contaminants

- ("vessel traffic" OR "boat traffic" OR "ship* traffic") AND (pollut* OR contamin* OR "water quality")

Vessel traffic and benthic sedimentation

- ("vessel traffic" OR "boat traffic" OR "ship* traffic") AND (benth* AND sediment*)

Vessel traffic and water clarity

- ("vessel traffic" OR "boat traffic" OR "ship* traffic") AND (turbid* OR "water clarity" OR light availab*)

Climate change and contaminants

- ("climate change" AND runoff AND (contaminant* OR pollut*) AND marine NOT tropic* NOT coral NOT *arctic)

Climate change and nutrients

- "climate change" AND nutrient* AND ("Salish Sea" OR "Puget Sound" OR "Strait of Georgia" OR "San Juan Islands" OR "Gulf Islands" OR "Vancouver Island" OR "Washington" OR "British Columbia")
- ("climate change" AND nutrient* AND marine NOT tropical NOT coral) categories: oceanography

Climate change and salinity

- "climate change" AND "salinity" AND marine AND ("Salish Sea" OR "Puget Sound" OR "Strait of Georgia" OR "San Juan Islands" OR "Gulf Islands" OR "Vancouver Island" OR "Washington" OR "British Columbia")
- ("climate change" AND "salinity" AND marine NOT tropical); categories: environmental sciences, oceanography

Climate change and benthic sedimentation

- "climate change" AND benthic AND sedimentation AND marine NOT tropic* NOT *arctic

Climate change and temperature

- "climate change" AND "temperature" AND ("Salish Sea" OR "Puget Sound" OR "Strait of Georgia" OR "San Juan Islands" OR "Gulf Islands" OR "Vancouver Island" OR "Washington" OR "British Columbia")

Climate change and water clarity

- ("climate change" AND (turbid* OR "water clarity" OR light availab*) AND marine NOT tropic*)

Shoreline development and nutrients

- "shoreline development" AND "nutrients"
- "shoreline development" AND "nitrogen"

Shoreline development and salinity

- "shoreline development" AND "salinity"
- shore* armor* salinity

Shoreline development and benthic sedimentation

- "shoreline development" AND "sediment"
- "shoreline armor*" AND "sediment"

Shoreline development and temperature

- "shoreline armor*" AND "temperature"

Pressures

Benthic sedimentation:

- kelp AND benthic AND sediment* AND marine NOT tropic*

Mechanical damage:

- kelp AND ("mechanical damage" OR damage OR graz* OR herb* OR defoliat*)

Epiphytes:

- kelp AND epiphyt*

Contaminants:

- kelp AND (pollut* OR contamin*) NOT tropic*

Nutrients

- "nutrient* AND (kelp or Laminariales)
- (nutrient* OR temperature) AND (kelp OR Laminariales)

Salinity

- kelp AND salinity

Water clarity

- kelp AND (turbid* OR "water clarity" OR light availab*)
